# Predictors of bacteremia and death, including immune status, in a large single-center cohort of unvaccinated ICU patients with COVID-19 pneumonia

**DOI:** 10.1186/s40001-023-01166-8

**Published:** 2023-07-03

**Authors:** Antonella Frattari, Ennio Polilli, Giorgia Rapacchiale, Simona Coladonato, Stefano Ianniruberto, Elena Mazzotta, Alessandro Patarchi, Mariangela Battilana, Raffaella Ciulli, Angelo Moretta, Lina Visocchi, Vincenzo Savini, Antonella Spacone, Rosamaria Zocaro, Fabrizio Carinci, Giustino Parruti

**Affiliations:** 1 Unit of Intensive Care, Pescara General Hospital, Pescara, Italy; 2 Clinical Pathology Unit, Pescara General Hospital, Pescara, Italy; 3 Infectious Diseases Unit, Pescara General Hospital, Pescara, Italy; 4 Microbiology and Virology Unit, Pescara General Hospital, Pescara, Italy; 5 Pneumology Unit, Pescara General Hospital, Pescara, Italy; 6 Department of Statistical Sciences, Università Di Bologna, Bologna, Italy

**Keywords:** Lymphocytopenia, Colonization, Herpesviridae, COVID-19, Bacteremia, ICU, Mortality

## Abstract

**Background:**

We investigated the possible role of the immune profile at ICU admission, among other well characterized clinical and laboratory predictors of unfavorable outcome in COVID-19 patients assisted in ICU.

**Methods:**

Retrospective analysis of clinical and laboratory data collected for all consecutive patients admitted to the ICUs of the General Hospital of Pescara (Abruzzo, Italy), between 1^st^ March 2020 and 30^th^ April 2021, with a confirmed diagnosis of COVID-19 respiratory failure. Logistic regressions were used to identify independent predictors of bacteremia and mortality.

**Results:**

Out of 431 patients included in the study, bacteremia was present in *N* = 191 (44.3%) and death occurred in *N* = 210 (48.7%). After multivariate analysis, increased risk of bacteremia was found for viral reactivation (OR = 3.28; 95% CI:1.83–6.08), pronation (3.36; 2.12–5.37) and orotracheal intubation (2.51; 1.58–4.02). Increased mortality was found for bacteremia (2.05; 1.31–3.22), viral reactivation (2.29; 1.29–4.19) and lymphocytes < 0.6 × 10^3^c/µL (2.32; 1.49–3.64).

**Conclusions:**

We found that viral reactivation, mostly due to Herpesviridae, was associated with increased risk of both bacteremia and mortality. In addition, pronation and intubation are strong predictors of bacteremia, which in turn together with severe lymphocytopenia due to SARS-CoV2 was associated with increased mortality. Most episodes of bacteremia, even due to Acinetobacter spp, were not predicted by microbiological evidence of colonization.

## Introduction

Prevention of catheter-associated and ventilator-associated infections, antimicrobial stewardship programs (ASP), as well as increased efficiency in microbiological support to early targeted antibiotic prescription, all represent bundle measures that may reduce the incidence and clinical impact of healthcare associated infections in acutely ill patients, at higher risk of both colonization and infection with Multidrug-Resistant Organisms (MDROs) [1–5]. MDRO colonization and bacteremia are common in immunocompetent patients with prolonged mechanical ventilation and ICU stay, and their clinical burden has been already investigated in unvaccinated patients with COVID-19 pneumonia and acute respiratory failure [6–9]. Early SARS-CoV2 pandemic waves represented an overwhelming challenge for healthcare professionals, and for intensivists in particular, as up to 20% of patients with SARS-CoV2 infection were admitted to the Intensive Care Unit (ICU) due to progression of respiratory failure, before vaccination and current therapeutic options [10, 11]. Such a high rate of admission to ICU, combined profound and persistent lymphocytopenia and frequent use of immunomodulatory drugs, e.g., steroids and tocilizumab [10, 11], put patients with COVID-19 pneumonia and acute respiratory failure at high risk of developing super-infections, including ventilator-associated pneumonia, bacterial or fungal bloodstream infections (BSIs), as well as massive reactivation of herpesviridae, with reports of increased morbidity and mortality during ICU stay [12–14].

Our local strategy to monitor and control both Gram-positive and Gram-negative MDRO colonizations was resumed soon after the first weeks in the pandemic. We observed a high proportion of COVID-19 ICU patients colonized by Alert and MDR bacteria across the first three pandemic waves, as well as a high proportion of ICU patients with bacteremia and herpes virus reactivation.

The aim of the present study was to evaluate the impact of immune status, including lymphocytopenia at ICU entrance, combined with the burden of MDRO colonization, on ensuing BSI and mortality in our large single center, consecutive cohort of patients with COVID-19 pneumonia.

## Materials and methods

### Study population

We performed a retrospective analysis of all consecutive patients admitted to the ICUs of the General Hospital of Pescara (Abruzzo, Italy) with a confirmed diagnosis of COVID-19, during the timeframe 1^st^ March 2020–30th April 2021. The diagnosis was confirmed through a swab test performed by the hospital personnel in the same occasion. The study was carried out in accordance with the Declaration of Helsinki (amended version). The local Health Administrative Board of the Pescara General Hospital (PGH) authorized and mandated daily collection of anonymized data for monitoring of local outcomes and research purposes, early in the health care emergency due to COVID-19.

The PGH is an urban 650-bedded tertiary facility of regional reference for adult traumas, acute diseases of neurosurgical interest, and COVID-19. The 3 early waves characterizing the epidemic in Italy during the reference period, required a different organization of ICU beds in the facility for patients affected by COVID-19. During the “first wave” (March–May 2020), patients were hosted either in a on purpose potentiated, pre-existing Infectious Diseases Ward, with 10 2-bedded rooms with negative pressure, or in a refurbished 7-bedded, open space ward with negative pressure. During the “second wave” (June–December 2020), patients were hosted in the 2 new, open space, 11- and 14-bedded ICU wards, built on purpose in the “COVID Hospital”, a separate building adjacent to and connected with the main tower of the hospital, both with negative pressure. During the “third wave” (January–April 2021), patients were distributed in the same 2 wards, as well as in a third, open space, 18-bedded ward in the same COVID Hospital, set up to cope with an overwhelming number of patients with acute respiratory failure. All wards were operated by an adequate number of ICU nurses, most of whom, however, were young and enrolled on purpose. Furthermore, all sites were overcrowded during most of the operation time. All patients were followed by the same group of intensivists, under a constant supervision. Daily rounds of interdisciplinary discussion were held for ICU patients most in need. Since the first wave, infection control measures, including dressing procedures, were recalled during working hours for fresh personnel, at least once in a month.

### Target characteristics

Lymphocyte subsets were assayed on the second or third day of ICU stay, based on judgement of the attending physician; IL6 and D-dimer were monitored each second day throughout ICU stay, as well as C-reactive protein, procalcitonin, complete blood counts, blood chemistry and coagulation parameters. Medical records of all patients in the ICUs were prospectively collected, including, age, sex, medical history, comorbidities, sepsis biomarkers and other laboratory parameters, invasive procedure delivered, including orotracheal intubation and cycles of pronation. The Charlson Comorbidity Index was calculated for all patients. All ICU patients were monitored for carriage of Staphylococci and Gram-negative MDR bacteria, both at entrance and weekly thereafter, using nasal and rectal swabs, and broncho-lavages. Blood cultures were drawn at each suspicion of sepsis or septic shock. Herpes virus reactivation (HSV1, HSV2, CMV, VZV, EBV, HHV6 and HHV8) was monitored on peripheral blood whenever suspected, due to worsening clinical conditions during ICU stay.

### Statistical analysis

The overall sample was used to investigate bacteremia and mortality at the ICU as primary outcomes. Candidate predictors included age as a continuous variable and a broad set of categorical variables, some of which using optimal cutoffs (Charlson index, CD4, lymphocytes, MDW, PCR, D-dimer, IL median) to provide better guidance for clinical interpretation and decision-making. A ROC analysis was performed using a separate 2 × 2 “confusion matrix” for every possible threshold applied to each continuous variable above [15]. The best threshold was defined by the highest value of the Youden Index [16].

The following candidate predictors were considered for mortality: waves of infection, diabetes, hypertension, obesity, bacteremia, colonization, viral reactivation, pronation, NIV, Charlson index, CD4, lymphocytes, MDW, PCR, D-dimer, IL median. Characteristics investigated for bacteremia included: age, gender, colonization, viral reactivation, pronation, IOT, Charlson index, lymphocytes, MDW, PCR, D-dimer.

Descriptive analysis included the calculation of absolute and relative frequencies for categorical variables and mean and standard deviation for age. Logistic regression was used for univariate and multivariate odds ratios of non-fatal outcomes [17]. An alpha level of 0.05 was used to present odds ratios together with their 95% confidence intervals and p values.

All predictive factors were included in a fully automated four-step backward elimination process of multivariate regression. Age (continuous) and gender were forced in all models, with all other variables sequentially excluded in three consecutive rounds using a *p* value ≥ 0.20, ≥ 0.10 and ≥ 0.05. All statistical analyses were carried out by developing ad hoc software in the R language [18].

## Results

A total of 2,159 patients were consecutively hospitalized at the PGH between March across the first three SARS-CoV2 pandemic waves in our district (March–May, 2020; October–December, 2020 and January–April, 2021), due to COVID-19 pneumonia. Among them, 442 patients were consecutively admitted in the Intensive Care Units, set up within Pescara General Hospital to tackle the unprecedented number of patients with acute respiratory failure due to COVID-19 pneumonia. The patients included in the cohort were distributed as follows: *N* = 96 were admitted during the first wave, hosted either in a on purpose potentiated, pre-existing Infectious Diseases Ward, with 10 2-bedded rooms with negative pressure (71 patients), or in a refurbished 7-bedded, open space ward with negative pressure (25 patients); *N* = 121 during the second wave, hosted in the 2 new, open space, 11- and 14-bedded ICU wards, on purpose built in the COVID Hospital, a separate building adjacent to and connected with the main tower of Pescara General Hospital, both with negative pressure; *N* = 245 during the third wave, admitted to the same 2 wards, as well as in a third, open space, 18-bedded ward in the same COVID Hospital, set up due to the overwhelming number of patients with acute respiratory failure. A total of 431 patients (97.5% of the sample) were included in the study population as they presented complete data collected for the main clinical characteristics.

The general characteristics of the study population are included in Table 1.

**Table 1:** General characteristics of the study population

Variable	Category	*N* (%)*
*N*		431
Age	Continuous	64.0 (12.2)
Gender	Female	132 (30.6)
	Male	299 (69.4)
Waves of infection	First	87 (20.2)
	Second	101 (23.4)
	Third	243 (56.4)
Diabetes	No	340 (78.9)
	Yes	91 (11.1)
Hypertension	No	274 (63.6)
	Yes	157 (36.1)
Obesity	No	354 (82.1)
	Yes	77 (17.9)
Bacteremia	No	240 (55.7)
	Yes	191 (44.3)
Colonization	No	262 (60.8)
	Yes	167 (39.2)
	Missing	2 ( 0.0)
Viral reactivation	No	350 (81.2)
	Yes	81 (18.8)
Pronation	No	192 (44.6)
	Yes	239 (55.4)
Orotracheal intubation (IOT)	No	171 (39.7)
	Yes	260 (60.3)
NIV	No	72 (16.7)
	Yes	248 (57.5)
	Missing	111 (25.8)
Bacteremia		
*Acinetobacter spp*	No	377 (87.4)
	Yes	54 (12.5)
*Klebsiella kpc*	No	416 (96.5)
	Yes	15 (3.5)
*Staphylococcus spp*	No	414 (96.0)
	Yes	17 (4.0)
*E. faecalis*	No	408 (94.6)
	Yes	23 (5.3)
*E. faecium*	No	394 (91.4)
	Yes	37 (8.6)
*Corynebacterium*	No	410 (95.1)
	Yes	21 (4.8)
Viral reactivation		
CMV	No	393 (91.1)
	Yes	38 (8.9)
HSV-1	No	395 (91.6)
	Yes	36 (8.4)
EBV	No	422 (97.9)
	Yes	9 (2.1)
Charlson Score Index	Continuous	2.8 (1.17)
CD4	Continuous	41.4 (12.6)
Lymphocytes	Continuous	0.8 (0.8)
Monocyte distribution width	Continuous	24.3 (4.1)
PCR	Continuous	108.4 (80.9)
D-dimer	Continuous	7.0 (17.2)
Median value of IL6	Continuous	207.0 (274.5)

*Mean, s.d. for continuous variables

Patients had a mean age of 64.0 years (s.d. = 12.2), with 69.4% males. In terms of comorbid conditions and risk factors, 91 (21.1%) were diabetic, 157 (36.4%) had hypertensive cardiopathy and 77 (17.9%) were obese. The average Charlson score was 2.8 ± 1.7, which did not vary across waves (2.7 ± 1.6; 3.0 ± 1.6 and 2.8 ± 1.8, p = 0.4, respectively, data not shown), suggesting a homogeneous distribution of comorbid conditions in the reference population. Severe lymphocytopenia (< 500 /mmc) was present in 103 patients (23.9%) at ICU entrance. The evaluation of lymphocyte subsets at ICU entrance was possible only in 277 (63%) patients, due to logistic constraints. One or more colonizing MDR organisms were isolated from 167 patients (38.7%).

At least one episode of bacteremia was recorded for 190 patients (44.1%). Indeed, most episodes of bacteremia were caused by bacteria not detected by routine microbiological monitoring, such as *Enterococcus faecium* or *faecalis* (37, 8.58%, and 23, 5.3%, respectively), *Corynebacterium* species (21, 4.8%) and *Staphylococcus* spp (17, %, Table 1). Furthermore, 81 patients had evidence of reactivation of any of the monitored herpes viruses: 36 (8.4%) HSV1, 38 (8.9%) CMV, 9 (2.1%) EBV. There were 54 (12.2%) episodes of bacteremia caused by *Acinetobacter* spp, although preceded by documented colonization by *Acinetobacter* spp only in 4 cases (7%). Differently from other recent reports, Klebsiella KPC accounted only for 16 (3.7%) of total blood-stream isolates.

Predictors of bacteremia and death were tested both through univariate and multivariate analyses.

Univariate results for bacteremia showed that viral reactivation (OR:5.59, 95% CI: 3.20–9.77), pronation (OR:4.47, 95% CI: 2.95–6.78) and orotracheal Intubation (OR:4.12, 95% CI: 2.69–6.31) were the only variables significantly associated with the event of interest (see Table 2). Episodes of bacteremia were not significantly associated with carriage of MDRO at single or multiple sites (OR: 1.32, 95% CI: 0.89–1.95, p = 0.16).

**Table 2:** Univariate analysis of selected predictors by levels of bacteremia

Variable	Category	Bacteremia			
		No	Yes	OR (95% C.I.)	*p* > *χ*^2^
*N*		240 (55.7)	191 (44.3)		
Age	Continuous	63.4 (13.4)	64.8 (10.5)	1.01 (0.99–1.03)	0.22
Gender	Female	78 (32.5)	54 (28.3)	1.00 (–)	
	Male	162 (67.5)	137 (71.7)	1.22 (0.81–1.85)	0.34
Colonization	No	153 (63.7)	109 (57.1)	1.00 (–)	
	Yes	86 (35.8)	81 (42.4)	1.32 (0.89–1.95)	0.16
	Missing	1 ( 0.4)	1 ( 0.5)		
Viral reactivation	No	221 (92.1)	129 (67.5)	1.00 (–)	
	Yes	19 ( 7.9)	62 (32.5)	**5.59 (3.20–9.77)**	** < 0.001**
Pronation	No	144 (60.0)	48 (25.1)	1.00 (–)	
	Yes	96 (40.0)	143 (74.9)	**4.47 (2.95–6.78)**	** < 0.001**
Orotracheal intubation (IOT)	No	129 (53.8)	42 (22.0)	1.00 (–)	
	Yes	111 (46.2)	149 (78.0)	**4.12 (2.69–6.31)**	** < 0.001**
Charlson Score Index	< 2	100 (41.7)	80 (41.9)	1.00 (–)	
	≥ 2	132 (55.0)	96 (50.3)	0.91 (0.61–1.35)	0.64
	Missing	8 ( 3.3)	15 (7.9)		
Lymphocytes	≥ 1.2 × 10^3^c/µL	149 (62.1)	115 (60.2)	1.00 (–)	
	< 1.2 × 10^3^c/µL	86 (35.8)	76 (39.8)	1.14 (0.77–1.70)	0.50
	Missing	5 ( 2.1)	0 (0.0)		
Monocyte distribution width	< 25	30 (12.5)	32 (16.8)	1.00 (–)	
	≥ 25	197 (82.1)	157 (82.2)	0.75 (0.44–1.28)	0.29
	Missing	13 ( 5.4)	2 (1.0)		
PCR	> 86 mg/mL	145 (60.4)	123 (64.4)	1.00 (–)	
	≥ 86 mg/mL	72 (30.0)	65 (34.0)	1.06 (0.70–1.61)	0.77
	Missing	23 ( 9.6)	3 (1.6)		
D-dimer	< 1 mg/mL	75 (31.2)	57 (29.8)	1.00 (–)	
	≥ 1 mg/mL	147 (61.3)	132 (69.1)	1.18 (0.78–1.79)	0.43
	Missing	18 ( 7.5)	2 (1.0)		

Significant ORs (*p* < 0.05) highlighted in bold

*n*,% or mean, s.d. for continuous variables

Univariate results for mortality are shown in Table 3. The strongest association was found for patients with viral reactivation (OR:3.28, 95% CI: 1.94–5.57) and Charlson Score Index ≥ 3 (OR:3.73, 95% CI: 2.46–5.64). Increased risk was also found for subjects for diabetes (OR:2.05, 95% CI: 1.27–3.30); bacteremia (OR:2.40, 95% CI: 1.63–3.55); CD4 ≥ 36 (OR:0.59, 95% CI:0.36–0.97); lymphocytes < 0.6 × 10^3^c/µL (OR:2.64, 95% CI: 1.77–3.96); PCR ≥ 134 mg/mL (OR:1.78, 95% CI: 1.17–2.71) and D-dimer ≥ 1 mg/mL (OR:2.22, 95% CI: 1.45–3.40). No association was found for the colonization with MDR bacteria (OR: 1.03, 95% CI: 0.70–1.51, *p* = 0.8).

**Table 3:** Univariate analysis of selected predictors by levels of mortality

Variable	Category	Mortality			
		No	Yes	OR (95% C.I.)	*p* > *χ*^2^
N		221 (51.3)	210 (48.7)		
Age	Continuous	60.0 (13.0)	68.1 ( 9.7)	1.07 (1.05–1.09)	< 0.001
Gender	Female	77 (34.8)	55 (26.2)	1.00 (–)	
	Male	144 (65.2)	155 (73.8)	1.51 (1.00–2.28)	0.051
Waves of infection	First	51 (23.0)	36 (17.1)	1.00 (–)	
	Second	45 (20.4)	56 (26.7)	1.42 (0.91–2.23)	0.122
	Third	125 (56.6)	118 (56.2)	0.99 (0.67–1.44)	0.938
Diabetes	No	187 (84.6)	153 (72.9)	1.00 (–)	
	Yes	34 (15.4)	57 (27.1)	**2.05 (1.27–3.30)**	** < 0.01**
Hypertension	No	146 (66.1)	128 (61.0)	1.00 (–)	
	Yes	75 (33.9)	82 (39.0)	1.25 (0.84–1.85)	0.270
Obesity	No	178 (80.5)	176 (83.8)	1.00 (–)	
	Yes	43 (19.5)	34 (16.2)	0.80 (0.49–1.31)	0.376
Bacteremia	No	146 (66.1)	94 (44.8)	1.00 (–)	
	Yes	75 (33.9)	116 (55.2)	**2.40 (1.63–3.55)**	** < 0.001**
Colonization	No	135 (61.1)	127 (60.5)	1.00 (–)	
	Yes	85 (38.5)	82 (39.0)	1.03 (0.70–1.51)	0.899
	Missing	1 ( 0.5)	1 ( 0.5)		
Viral reactivation	No	198 (89.6)	152 (72.4)	1.00 (–)	
	Yes	23 (10.4)	58 (27.6)	**3.28 (1.94–5.57)**	** < 0.001**
Pronation	No	96 (43.4)	96 (45.7)	1.00 (–)	
	Yes	125 (56.6)	114 (54.3)	0.91 (0.62–1.33)	0.635
NIV	No	31 (14.0)	41 (19.5)	1.00 (–)	
	Yes	128 (57.9)	120 (57.1)	0.71 (0.42–1.20)	0.200
	Missing	62 (28.1)	49 (23.3)		
Charlson Score Index	< 2	124 (56.1)	56 (26.7)	1.00 (–)	
	≥ 3	85 (38.5)	143 (68.1)	**3.73 (2.46–5.64)**	** < 0.001**
	Missing	12 ( 5.4)	11 ( 5.2)		
CD4	< 36	41 (18.6)	58 (27.6)	1.00 (–)	
	≥ 36	97 (43.9)	81 (38.6)	**0.59 (0.36–0.97)**	**0.037**
	Missing	83 (37.6)	71 (33.8)		
Lymphocytes	≥ 0.6 × 10^3^c/µL	159 (71.9)	105 (50.0)	1.00 (–)	
	< 0.6 × 10^3^c/µL	59 (26.7)	103 (49.0)	**2.64 (1.77–3.96)**	** < 0.001**
	Missing	3 ( 1.4)	2 ( 1.0)		
Monocyte distribution width	< 20	30 (13.6)	32 (15.2)	1.00 (–)	
	≥ 20	186 (84.2)	168 (80.0)	0.85 (0.49–1.45)	0.546
	Missing	5 ( 2.3)	10 ( 4.8)		
PCR	> 134 mg/mL	146 (66.1)	122 (58.1)	1.00 (–)	
	≥ 134 mg/mL	55 (24.9)	82 (39.0)	**1.78 (1.17–2.71)**	** < 0.01**
	Missing	20 ( 9.0)	6 ( 2.9)		
D-dimer	< 1 mg/mL	84 (38.0)	48 (22.9)	1.00 (–)	
	≥ 1 mg/mL	123 (55.7)	156 (74.3)	**2.22 (1.45–3.40)**	
	Missing	14 (6.3)	6 (2.8)		
Median value of IL6	< 28 mg/mL	33 (14.9)	23 (11.0)	1.00 (–)	
	≥ 28 mg/mL	139 (62.9)	156 (74.3)	1.61 (0.90–2.87)	0.1
	Missing	49 (22.2)	31 (14.8)		

Significant ORs (*p* < 0.05) highlighted in bold

*n*,% or mean, s.d. for continuous variables

Multivariate results for bacteremia and mortality are presented in Table 4. Episodes of bacteremia were significantly associated with intubation (OR:2.5, 95% CI: 1.58–4.02, *p* = 0.001), pronation cycles (OR:3.36, 95% CI: 2.12–5.37, *p* < 0.0001) and reactivation of any Herpes virus (OR:3.28, 95% CI: 1.83–6.08, *p* = 0.001).

**Table 4:** Results of multivariate regression for bacteremia and mortality

Variable	Category	Bacteremia		Mortality	
		OR (95% C.I.)	p > χ^2^	OR (95% C.I.)	p > χ^2^
Age	Continuous	1.01 (0.99–1.03)	0.1769	1.07 (1.05–1.09)	** < 0.001**
Gender	Female	1.00 (–)		1.00 (–)	
	Male	1.07 (0.67–1.71)	0.7771	1.55 (0.97–2.49)	0.0711
Bacteremia	No			1.00 (–)	
	Yes			**2.05 (1.31–3.22)**	** < 0.001**
Viral reactivation	No	1.00 (–)		1.00 (–)	
	Yes	**3.28 (1.83–6.08)**	< 0.001	**2.29 (1.29–4.19)**	** < 0.01**
Pronation	No	1.00 (–)			
	Yes	**3.36 (2.12–5.37)**	< 0.001		
Orotracheal intubation (IOT)	No	1.00 (–)			
	Yes	**2.51 (1.58–4.02)**	< 0.001		
Lymphocytes	≥ 0.6 × 10^3^c/µL			1.00 (–)	
	< 0.6 × 10^3^c/µL			**2.32 (1.49–3.64)**	** < 0.001**

Significant ORs (*p* < 0.05) highlighted in bold

After multivariate analysis, significantly increased risk of mortality was found for lymphocytopenia (OR:2.32, 95% CI: 1.49–3.64, *p* = 0.0002), ensuing bacteremia (OR: 2.05, 95% CI: 1.31–3.22, *p* = 0.0014) and reactivation of herpes viruses (OR: 2.29, 95% CI: 1.29–4.19, *p* = 0.0057).

Evaluation of individual lymphocyte subsets as possible predictors of both bacteremia and death available in 277 (63%) patients were not found significant. Similarly, first, second or third waves of infection did not enter in the univariate or final multivariate analyses because non-significantly associated with any considered outcome.

## Discussion

Recent literature reports clearly pointed out that increasing age and relevant comorbidities as diabetes, hypertension and obesity represented an array of risk factors for unfavorable outcome in COVID-19 patients with progression of respiratory failure in the ICU [19]. A few studies also reported MDRO carriage as a potential risk factor for ensuing bacteremia and mortality [20].

Since the beginning of the first pandemic wave, we were interested in gathering information on the possible role of immune depression induced by SARS CoV2 infection. As a consequence, in the search for more predictors to define patients’ prognosis, we systematically monitored all relevant immunological parameters of our patients. Consistently with our previous experiences and the recent literature, we collected at ICU admission daily lymphocyte counts and assays of lymphocyte subpopulations and Ig classes [21–23]. We also monitored herpetic reactivation as a possible consequence of SARS CoV2-related immune imbalance, finding it to be more frequent than initially expected, based on the available literature, particularly among patients with lymphocytes persistently below 400/mmc [24]. Beyond these purposes, we tried to keep quite high our focus on prevention of MDRO colonizations and infections, screening MDROs at ICU entry and weekly along ICU stay [25]. Indeed, a remarkable proportion of ICU patients in our series presented one or more colonizing MDRO, in line with several other reports in the recent literature, witnessing MDRO carriage in 33% of ICU patients with COVID-19 pneumonia, as management of COVID-19 patients posed serious operational challenges in our as in others’ settings [26]. Our Microbiology Unit was overburdened, with a reduction in efficiency for early microbiological characterization of patients. In addition, we had few or no chances of isolating patients with documented carriage of MDROs. Working with suits and helmets hampered movements and maneuvers, and the limited time spent by nurses on each patient resulted into potentially decreased attention on infection control measures [27]. In addition, although antibiotic stewardship programs were kept in place, concerns surrounding the outcome of COVID-19 patients with clinical deterioration pushed clinicians to frequently prescribe antibiotics [28]. We therefore hypothesized that increased carriage and possible horizontal transmission of colonizing MDROs might be linked to the frequent episodes of ensuing bacteremia, observed in our series as in others’, whose impact on patients’ prognosis is well known [29]. At odds with our fears, both univariate and multivariate statistical models reveled that carriage of MDROs, although quite frequent in our series, did not translate in and was not significantly linked with an increased risk of bacteremia during ICU stay.

Significantly higher rates of bacteremia were instead associated with repeated cycles of pronation and with prolonged sedation for orotracheal intubation. This could be likely due to an increased bacterial translocation in the gut of prone patients, a phenomenon that has been already reported by others in COVID-19 [30]. In fact, SARS-CoV-2-associated coagulopathy may affect both micro- and macrocirculation of the intestine, increasing the risk of bacterial translocation out of the gastrointestinal tract, together with endothelial dysfunctions of the digestive tract, frequently observed in such patients [31]. This could be particularly true for bacterial species that are normal bulky constituents of gut microbiota, such as Enterococci and Corynebacteria, with unremarkable antibiotic resistance profiles. These factors, however, were found to be associated also with bacteremias by Acinetobacter species. In fact, a total of 56 episodes of Acinetobacter bacteremia occurred during ICU stay involved patients with persistently low absolute lymphocyte counts and high IL6- levels, of which only 4 (7%) were ushered by previous evidence of colonization.

Our results do not obviously encourage the reduction of infection control measures, which are indispensable to guarantee the cautious delivery of ICU care to COVID-19 patients [32]. The management of COVID-19-induced pneumonia and associated acute respiratory failure, however, appears still to be a relatively uncharted territory, despite recent efforts to outline a comprehensive picture, including targeted preventative and therapeutic measures [32]. Therefore, our results suggest that ordinary measures to control ensuing bacteremia and the consequent increased risk of death are yet insufficient to reduce the actual risk of ensuing bacteremia in the setting of COVID-19 critical patients. These results may partly be explained by the evidence of independent prediction of unfavorable outcomes by lymphocytopenia in unvaccinated patients with SARS CoV2 infection, which may ease bacterial translocation from the gut in its turn [33].

The entity of Herpesviridae reactivation in our series, systematically searched on peripheral blood cells as a complement of immune characterization, appeared to be somewhat surprising, especially in terms of ability to predict mortality independently [34]. Search for Herpesviridae reactivation also on broncho-alveolar lavages might have yielded even more relevant results. So, further studies may be hypothesized, including systematic collection of respiratory samples, together with early access to acyclovir, which might potentially yield reduction of morbidity and mortality in such patients [35].

As it is known, SARS CoV-2 produced different variants along the considered timeframe. A random sampling of genetic screening to monitor the emergence of new variants over time was implemented by our regional health system. Thus, on the basis of these surveillance systems, it is well acknowledged that in our regional area the most prevalent variants during the first and second/third waves were, respectively, the wild-type SARS CoV-2 with 98% of cases and the Alpha SARS CoV-2 with 96% of cases. However, as mentioned in the result section, both bacteremia and death were not influenced by the waves of infection.

Our study has several strengths and limitations that are worth outlining.

As far as strengths are concerned, with its prospective design, our study allowed to collect data from over 95% cases admitted to the ICU. Furthermore, the immune status of our patients was well characterized, using frequent absolute lymphocyte counts in all patients, as well as lymphocyte subset assays and immunoglobulin dosages in approximately two-thirds of our cohort. Selection bias can be ruled out for this subsample, as assays of lymphocyte subsets were missed in the remaining patients only due to laboratory constraints. Finally, microbiological characterization was systematically performed across the three study periods for all patients.

The main limitation of our study was that it included only a single center for data collection. Further, the observations have been carried out in a period antecedent to the introduction of massive vaccination to control pathogenesis and lethality of SARS CoV2. This may limit the applicability of our findings to those patients admitted to the ICU worldwide, who are still not vaccinated.

## Conclusions

Our investigation, performed on a large sample of unvaccinated patients with SARS CoV2 pneumonia admitted to ICU in a single Italian center due to respiratory failure, provides evidence that severe lymphocytopenia due to SARS-CoV2 may associate with ensuing bacteremia and Herpesviridiae reactivation in a large proportion of cases, independently predicting mortality. Most episodes of bacteremia, even due to Acinetobacter spp, were not predicted by microbiological evidence of MDR colonization, suggesting a major role for pronation cycles and prolonged sedation in easing blood translocation of gut bacteria undetected by routine microbiological monitoring. Our results also reveal an unexpectedly high proportion of Herpesviridae reactivation in our settings, heralding an unfavorable outcome in unvaccinated critical patients with COVID-19 pneumonia.

## Data Availability

Data analyzed in the current study are available from the corresponding author on reasonable request.
